# Supplementary material for: A Rhodopseudomonas strain with a substantially smaller genome retains the core metabolic versatility of its genus
Source: Appl Environ Microbiol. 2025 Mar 10;91(4):e02056-24. doi: 10.1128/aem.02056-24 (PMC12016538; doi:10.1128/aem.02056-24)
Supplement: Supplemental legends — Legends for supplemental files. [file aem.02056-24-s0003.docx]

**SUPPLEMENTAL MATERIAL LEGENDS**

**FIG. S1.** Phylogenetic relationships of *Rhodopseudomonas* strains based on 16S rRNA gene sequences. Bootstrap values (100 replicates) are given at branch points (only showing values of > 50). Bar represents substitutions per site. *Bradyrhizobium diazoefficiens* USDA110 was used to root the tree.

**FIG. S2.** COG category analysis of DSM127 against 37 other *Rhodopseudomonas* strains. COG categories: C, Energy production and conversion; D, Cell cycle control, cell division, chromosome partitioning; E, Amino acid transport and metabolism; F, Nucleotide transport and metabolism; G, Carbohydrate transport and metabolism; H, Coenzyme transport and metabolism; I, Lipid transport and metabolism; J, Translation, ribosomal structure and biogenesis; K, Transcription; L, Replication, recombination and repair; M, Cell wall/membrane/envelope biogenesis; N, Cell motility; O, Posttranslational modification, protein turnover, chaperones; P, Inorganic ion transport and metabolism; Q, Secondary metabolites biosynthesis, transport and catabolism; R, General function prediction only; S, Function unknown; T, Signal transduction mechanisms; U, Intracellular trafficking, secretion, and vesicular transport; V, Defense mechanisms. For this figure only, we first calculated the average number of gene counts for each COG using our 37 *Rhodopseudomonas* strain set (excluding DSM127) (Table S1) and compared with the gene count from DSM127. Those COGs that had an average count of less than one were excluded. See Table S4 for detailed COG analysis of each strain.

**TABLE S1.** Reference genome sequences used in this study (supplied as an Excel file).

**TABLE S2.** Gene inventory comparisons of *Rhodopseudomonas* strains CGA009 and DSM127 (supplied as an Excel file).

**TABLE S3.** Gene products and locus tags of some of the DSM127 genes described in this study (supplied as an Excel file).

**TABLE S4.** A COG analysis of *Rhodopseudomonas* strains (supplied as an Excel file).

**TABLE S5.** Carbon source use by *Rhodopseudomonas* strains CGA009 and DSM127 (supplied as an Excel file).
